# Use of androgen suppression therapy at salvage radiotherapy in recurrent prostate cancer: a systematic review with meta-analysis

**DOI:** 10.3389/fruro.2026.1836469

**Published:** 2026-08-17

**Authors:** Maria Juliana Chaves Medina, Rodolfo Varela Ramírez, Wilfredo Donoso Donoso, Jaime Moreno-Chaparro, Javier Eslava-Schmalbach

**Affiliations:** 1 Urology Research and Innovation Group, Faculty of Medicine, Universidad Nacional de Colombia, Bogotá, Colombia; 2 Hospital Universitario Nacional de Colombia, Bogotá, Colombia; 3 Urology Unit, Instituto Nacional de Cancerología, Bogotá, Colombia; 4 Urology Unit, Department of Surgery, Faculty of Medicine, Universidad Nacional de Colombia, Bogotá, Colombia; 5 Department of Clinical Epidemiology and Biostatistics, Faculty of Medicine, Universidad Nacional de Colombia, Bogotá, Colombia

**Keywords:** androgen antagonists, gonadotropin-releasing hormone, neoplasm recurrence local, prostatectomy, radiotherapy, salvage therapy

## Abstract

**Introduction:**

Salvage radiotherapy (SRT) is one of the treatment options after progression following surgical management, with effectiveness varying according to patient risk and clinical-pathological characteristics.

**Objective:**

To compare the effectiveness of SRT alone versus SRT combined with androgen deprivation therapy (ADT) in men with biochemical recurrence.

**Methods:**

We conducted a systematic review with advanced searches in MEDLINE (PubMed), EMBASE, SCOPUS, Cochrane CENTRAL, and LILACS for the period 1990–2024. Two independent reviewers performed study selection (titles/abstracts and full text) and data extraction. Risk of bias was assessed with ROBINS-I and RoB 2.0. A random-effects meta-analysis was used to pool effect estimates. PROSPERO registration: CRD42025640604.

**Results:**

Thirteen studies were included. Nine reported overall survival (OS) and biochemical progression-free survival (b-PFS) respectively, four clinical progression-free survival (c-PFS), and eight metastasis-free survival (MFS). Combined SRT+ADT was associated with improved b-PFS (HR 0.51; 95% CI 0.45-0.57; I²=0%) and MFS (HR 0.71; 95% CI 0.60-0.84; I²=39%), whereas the remaining meta-analyzed outcomes showed no consistent benefit or were limited by heterogeneity. Regarding toxicity, the most frequent adverse events were endocrine and sexual, related to hormonal blockade, gynecomastia and erectile dysfunction.

**Conclusions:**

Combined SRT + ADT improves biochemical disease control and may reduce the risk of metastasis in selected patients with biochemical recurrence after radical postatectomy, whereas the effect on overall survival remains uncertain. Individualized management requires risk stratification when deciding on the addition and duration of ADT to optimize oncologic outcomes.

**Systematic review registration:**

https://www.crd.york.ac.uk/PROSPERO/view/, identifier CRD42025640604.

## 1 Introduction

Prostate cancer is the second most frequently diagnosed malignancy and the sixth leading cause of cancer-related death among men worldwide, with an estimated 1.4 million new cases and 375,000 deaths in 2020 (1, 2). Despite advances in early detection and radical surgery, up to 30% of patients are estimated to experience PSA persistence or biochemical recurrence following surgery (3), a clinically relevant event associated with subsequent clinical progression and potential metastatic disease (4).

Salvage radiotherapy (SRT) is considered a potentially curative option for patients with biochemical recurrence after radical prostatectomy (5). Retrospective studies suggest that an initial observation strategy, followed by radiotherapy combined with hormone therapy at the first sign of recurrence, may achieve progression-free survival (PFS) rates of approximately 64% (6, 7). However, adding hormone therapy may constitute overtreatment for some patients and is associated with higher costs and an increased risk of short- and long-term adverse effects, reported in up to 35% of cases (7).

The effectiveness of SRT varies according to prognostic factors. Pre-treatment PSA is a key determinant: benefit has been observed in patients with PSA levels < 1.5 ng/mL, whereas in those with PSA levels ≤ 0.6 ng/mL, a favorable benefit profile was not demonstrated, and treatment was instead associated with a higher risk of toxicity (8). Additional independent predictors of biochemical recurrence include extracapsular extension (pT3a), seminal vesicle invasion (pT3b), Gleason score > 8, and positive surgical margins (9, 10).

Several studies have reported improved outcomes with the combination of SRT and androgen deprivation therapy (ADT), with the largest effects observed in long-term PFS (10-year follow-up), particularly among patients with unfavorable prognostic features, where tumor control rates of up to 75% have been described (5). Clinically, the benefit of combined therapy appears in the long term, reducing biochemical recurrence and the risk of metastases (7).

Nevertheless, uncertainty remains regarding which patients derive the greatest benefit from ADT, the optimal timing of initiation, and the appropriate duration needed to maximize oncologic outcomes while minimizing toxicity and preserving quality of life (8, 11). Therefore, a comparative assessment of SRT alone versus SRT combined with ADT in patients with localized prostate cancer and biochemical recurrence (PSA ≥ 0.2 ng/mL) is warranted (12).

This systematic review and meta-analysis aimed to compare the effectiveness (both survival outcomes and toxicity) of salvage radiotherapy combined with hormone therapy versus salvage radiotherapy alone in patients with localized prostate cancer who develop biochemical recurrence after radical prostatectomy.

## 2 Methodology

### 2.1 Study design

We conducted a systematic literature review and meta-analysis in accordance with the *Cochrane Handbook for Systematic Reviews* (13) and the *Preferred Reporting Items for Systematic Reviews and Meta-Analyses (PRISMA)* guidelines (14). The study protocol was prospectively registered in the International Prospective Register of Systematic Reviews (PROSPERO); registration number CRD42025640604.

### 2.2 Eligibility criteria

- Population: Men aged ≥18 years with histologically confirmed prostate adenocarcinoma who underwent radical prostatectomy with curative intent for localized disease and subsequently developed biochemical recurrence (PSA ≥0.2 ng/mL).
- Intervention: Salvage radiotherapy combined with hormone therapy (androgen deprivation therapy, ADT).
- Comparator: Salvage radiotherapy alone.
- Outcomes:
          ◦    Primary outcomes: Survival outcomes, including overall survival, clinical and biochemical progression-free survival, and metastasis-free survival.
                  •    Clinical Progression-free survival. Strictly restricted to objective, radiographically or pathologically documented local, regional, or distant recurrence, excluding isolated PSA rises.
                  •    Biochemical Progression-free survival. Defined and commonly used as a PSA level ≥ 0.2 ng/mL confirmed by two separate measurements.
                  •    Metastasis-free survival. Evaluated as a distinct composite endpoint, defined as the time from randomization to the first documentation of distant metastasis (visceral or bone disease) or death from any cause.
          ◦    Secondary outcomes: Adverse events (toxicity).
- Study designs: Randomized controlled trials, cohort studies, and case-control studies.
- Exclusion criteria: Studies including patients with metastatic disease (M1), prior pelvic radiotherapy, prior ADT, or those undergoing salvage radical prostatectomy.

### 2.3 Electronic literature search

We performed a systematic literature search in MEDLINE (PubMed), EMBASE, Scopus, LILACS, and the Cochrane Central Register of Controlled Trials (CENTRAL). Additional potentially eligible studies were identified through Google Scholar (first five pages of results). Searches were limited to publications in English or Spanish and to the period from January 1990 through December 2024. The full search strategy and database-specific terms are provided in Supplementary Appendix 1.

### 2.4 Study selection and data extraction

Two reviewers independently screened titles and abstracts and then assessed the full text of potentially eligible studies against the predefined eligibility criteria. Disagreements were resolved by consensus. Data extraction was performed independently by two reviewers using a standardized, pre-specified data collection form.

### 2.5 Risk of bias assessment

The risk of bias in observational studies was assessed using the ROBINS-I tool (15). Randomized controlled trials were evaluated using the Cochrane Risk of Bias tool, RoB 2.0 (16).

### 2.6 Data synthesis

Data were summarized descriptively, distinguishing primary outcomes (survival) from secondary outcomes (toxicity). We present a flow diagram depicting the study selection process and tables summarizing baseline characteristics and key outcomes of the included studies.

### 2.7 Statistical analysis

We performed a primary synthesis of time-to-event outcomes as reported by the included studies (overall survival (OS), clinical and/or biochemical progression-free survival (PFS), and metastasis-free survival (MFS)), using hazard ratios (HRs) with 95% confidence intervals (CIs). Analysis was restricted to studies that were comparable in design and outcome definitions. For toxicity, the primary endpoint was the proportion of adverse events of grade ≥2-3; results were summarized descriptively and stratified by intervention group.

For the meta-analysis, we used hazard ratios (HRs) and 95% confidence intervals as reported in the original studies and pooled estimates using a random-effects model to account for expected between-study variability (17). We conducted prespecified subgroup analyses by study design (randomized controlled trials vs. observational studies) to evaluate the consistency of results; the primary analysis pooled evidence across study designs in a single meta-analysis (18). Statistical heterogeneity was quantified using the I² statistic, which estimates the proportion of total variability attributable to between-study heterogeneity. Publication bias was assessed visually using funnel plots. All analyses were performed using Review Manager (RevMan) version 5.4 (19).

## 3 Results

**Figure 1:**
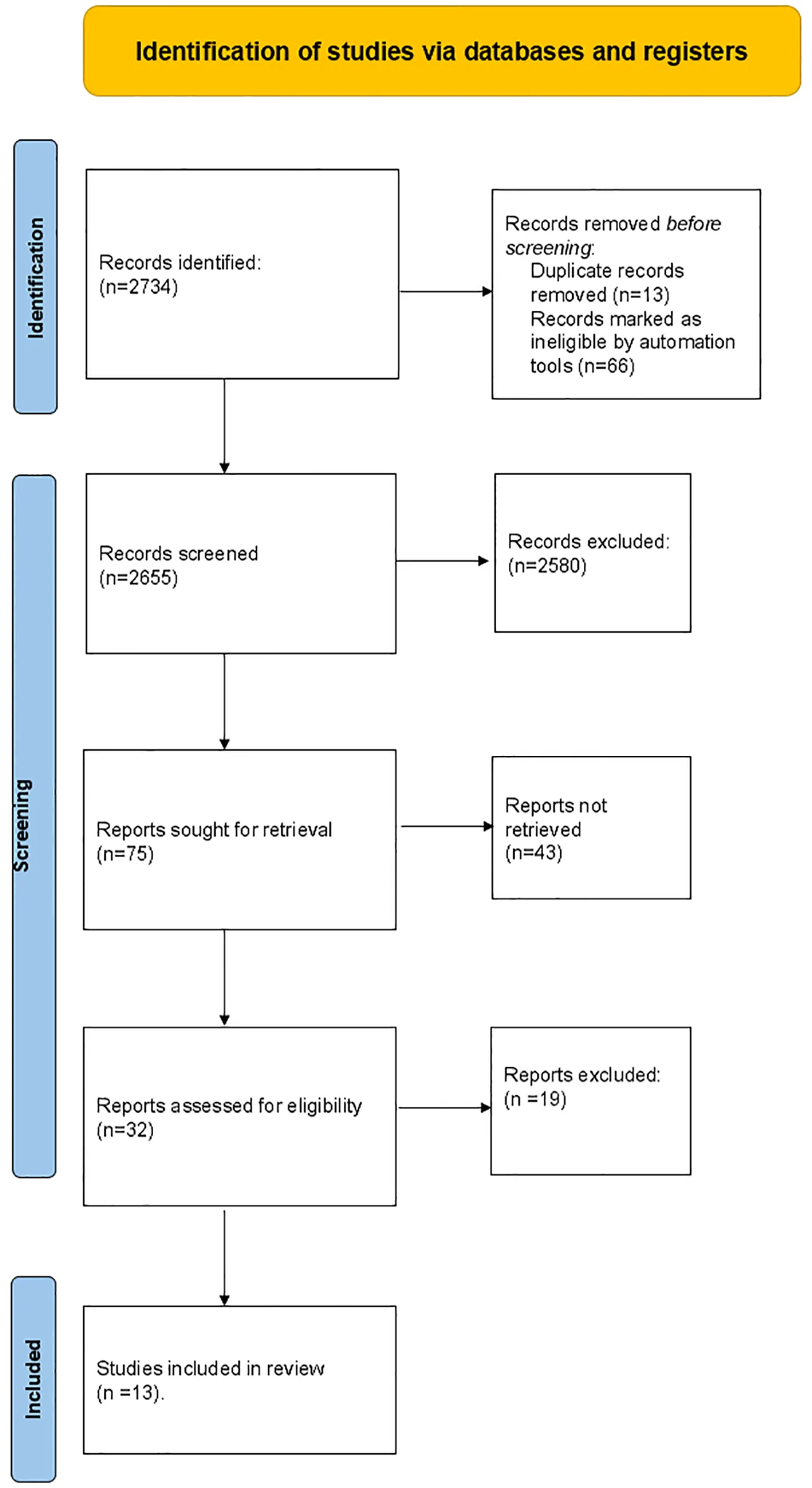
PRISMA flow diagram.

The literature search identified 2,734 records. After removing 13 duplicates and 66 records deemed ineligible during initial deduplication/cleaning, 2,655 records were screened by title and abstract, and 2,580 were excluded. Seventy-five records underwent further assessment, of which 43 were excluded. Full-text eligibility was assessed for 32 articles; 19 were excluded, and 13 studies were included [**Figure 1**; (20)].

### 3.1 Characteristics of the included studies

Thirteen studies published between 2006 and 2024 were included (**Table 1**). Seven were randomized clinical trials: Shipley et al. (2017) (21), Carrie et al. (2019) (22), Yokomizo et al. (2019) (23), Pollack et al. (2022) (24), Elakshar et al. (2023) (25), Parker et al. (2024) (26, 27). Additionally, six observational studies were included: Ospino et al. (2012) (28), Cortés-González et al. (2013) (29), Ervandian et al. (2016) (30), Tendulkar et al. (2016) (31), Yoo et al. (2017) (32) and Fossati et al. (2019) (33).

**Table 1:** Characteristics of the included studies.

Author	Year	Country	Study design	Sample size (n)	Risk factors	% with ADT
Ospino et Al.	2012	Colombia	Retrospective cohort	40	PSA pre-SRT >1 ng/mL, positive margins.	22
Cortés-González et Al.	2013	Sweden	Retrospective cohort	184	PSA pre-SRT >2 ng/mL, pT≥3b, Gleason ≥8, SVI	100
Ervandian et Al.	2016	Denmark	Retrospective cohort	259	Positive margins, PSA pre-SRT >0,5 ng/ml.	44
Tendulkar et Al.	2016	United States	Retrospective cohort	2460	Pre-SRT PSA >2,0 ng/mL, Gleason ≥8, SVI, positive margins.	22
Shipley et Al.	2017	United States	Phase III RCT	760	PSA ≥1,5 ng/mL, Gleason ≥8, positive margins.	100
Yoo et Al.	2017	South Korea	Retrospective cohort	162	PSA pre-SRT ≥0,6 ng/mL, pT≥3b	42
Fossati et Al.	2019	Italy, United States, France, Germany, Austria, Belgium	Retrospective cohort	1264	pT≥3b, Gleason ≥8, PSA pre-SRT >0,5 ng/mL	29
Carrie et Al.	2019	France	Phase III RCT	743	pT≥3b, Gleason ≥8, positive margins.	100
Yokomizo et Al.	2019	Japan	Phase III RCT	210	Positive margins, time from RP to SRT >12m.	100
Pollack et Al.	2022	United States, Canada, Israel	Phase III RCT	1716	PSA pre-SRT > 1,0 ng/mL, Gleason ≥ 8, pT≥3b, positive margins, PSA-DT ≤ 6 m.	66
Elakshar et Al.	2023	Canada	Phase II RCT	46	Gleason ≥8, pT≥3, PSA pre-SRT >20 ng/mL, N1	100
Parker et Al.	2024	Canada, Denmark, Ireland, United Kingdom	Phase III RCT	1523	SRT: 57% Gleason ≥ 8, positive margins, pT≥3b, SVI	100
Parker et Al.	2024	Canada, Denmark, Ireland, United Kingdom	Phase III RCT	1480	SRT: 71%, PSA pre-RT ≥0,5 ng/mL, ≥pT3, ISUP 4–5 (Gleason ≥ 8), SVI.	50

ADT, androgen deprivation therapy; RCT, randomized controlled trial; PSA, prostate-specific antigen; RP, radical prostatectomy; SRT, salvage radiotherapy; PSA-DT, PSA doubling time; pT, pathologic stage; SVI, seminal vesicle invasion.

Studies were geographically diverse, spanning multiple continents. Three studies were conducted in North America (21, 25, 31) and three in Europe (22, 29, 30); two in Asia (23, 32), and one in South America (28). Four studies were multicenter (24, 26, 27, 33).

Overall, 10,847 patients were included, with broadly similar baseline characteristics and a mean age of 65 years. All patients had previously undergone radical prostatectomy and received salvage radiotherapy (SRT). Across the randomized trials, ≥90% of participants had a pre-SRT PSA <2.0 ng/mL. Gleason score 7 was the most common histopathologic grade (68%). Approximately 35% of patients across the included trials and cohorts were classified as high risk; Gleason score ≥8 was reported in 30%, and pathologic stage ≥pT3 in 50%.

Regarding hormone therapy, its use across studies averaged 67%, with regimens ranging from short-term courses of 6 months to extended schedules of up to 24 months. The interval between radical prostatectomy and initiation of SRT ranged from 12 to 36 months.

For the primary outcomes, nine studies reported overall survival (OS) (21–27, 29, 32). Biochemical and/or clinical progression-free survival (PFS) was reported across most included studies (21–27, 29–33). Metastasis-free survival (MFS) was reported in eight studies (21, 22, 24–27, 31, 32).

Toxicity outcomes were reported in eleven studies (21–30, 32). Gastrointestinal and genitourinary adverse events were more frequent. Most adverse events were endocrine and sexual in nature and were primarily attributable to hormone therapy, with gynecomastia occurring at a high frequency among patients receiving ADT.

### 3.2 Survival

#### 3.2.1 Overall survival

Regarding OS, Shipley et al. (2017) (21) demonstrated a clear benefit of combined therapy, confined to patients with high-risk features (PSA ≥1.5 ng/mL, Gleason score ≥8, or positive surgical margins), corresponding to a 23% relative reduction in the hazard of death (12-year OS: 76.3% vs 71.3%; HR 0.77; 95% CI 0.59–0.99; p=0.04). In contrast, Yokomizo et al. (2019) (23), Carrie et al. (2019) (22), Yoo et al. (2017) (32), Pollack et al. (2022) (24) and Parker et al. (2024) (27), reported no statistically significant differences in OS at 5–10 years between SRT alone and combined therapy. Two observational studies Elakshar et al. (2023) (25) and Cortés-González et al. (2013) (29), reported high OS rates (95% and 97% at 4 and 10 years, respectively) among patients receiving combined therapy; however, given the absence of direct comparators and adjusted effect estimates, these findings reflect favorable cohort prognosis rather than a demonstrated causal effect of ADT on OS.

**Figure 2:**
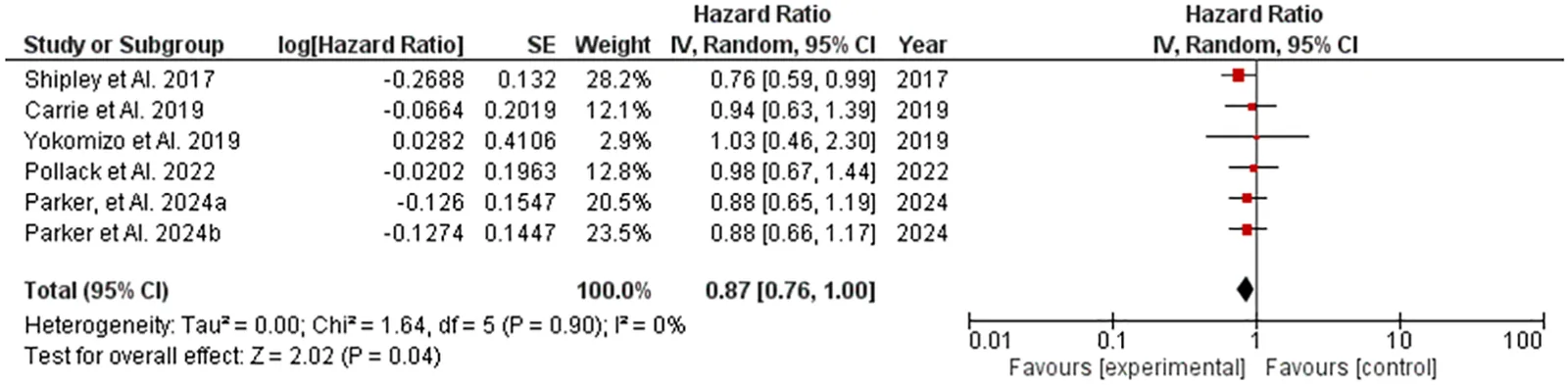
Meta-analysis of overall survival.

In the meta-analysis of studies reporting HRs (**Figure 2**), the addition of ADT to SRT yielded a pooled HR of 0.87 (95% CI 0.76–1.00; p=0.04; I²=0%), indicating a potentially modest reduction in mortality risk. Nevertheless, because the upper bound of the confidence interval reaches 1.00, the data are also compatible with no effect.

#### 3.2.2 Progression-free survival

When interpreting PFS, it is important to differentiate clinical PFS (c-PFS), which includes local recurrence, distant recurrence, or death from biochemical PFS (b-PFS), which is defined solely by PSA rise (34).

**Figure 3:**
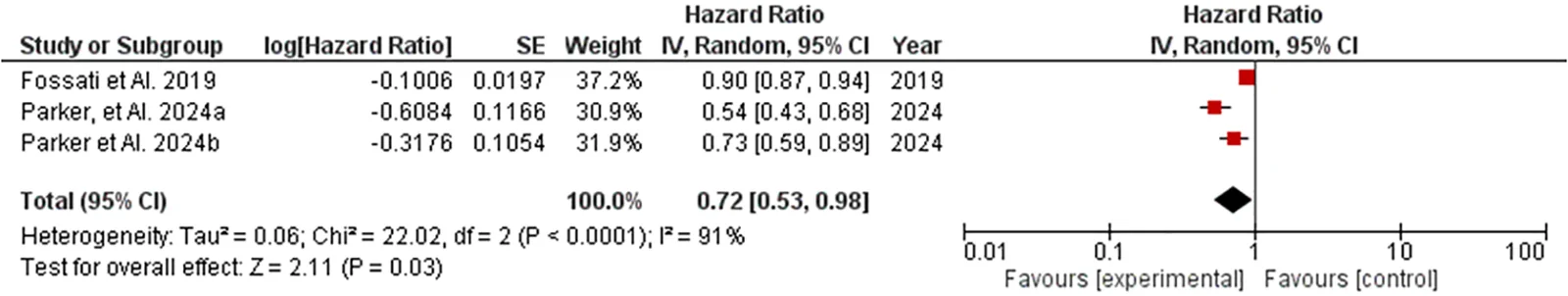
Meta-analysis of clinical progression-free survival.

Randomized evidence consistently indicates that adding ADT to SRT improves clinical progression-free survival (c-PFS). Carrie et al. (2019) (22), Parker et al. (2024) (26, 27) and Shipley et al. (2017) (21), reported a 25%-50% reduction in the hazard of clinical progression (HR 0.5-0.7), translating into absolute gains of approximately 10%-15% in c-PFS at 5–10 years. In line with these findings, the Fossati et al. (2019) observational cohort (33), suggested improved clinical control with extended ADT (36 months) among patients with high-risk features. In the corresponding meta-analysis (**Figure 3**), the addition of ADT to SRT was associated with a 28% relative reduction in the risk of progression or death (HR 0.72; 95% CI 0.53-0.98; p=0.03). Nonetheless, heterogeneity was substantial (I²=91%), indicating considerable between-study variability in the magnitude of benefit.

**Figure 4:**
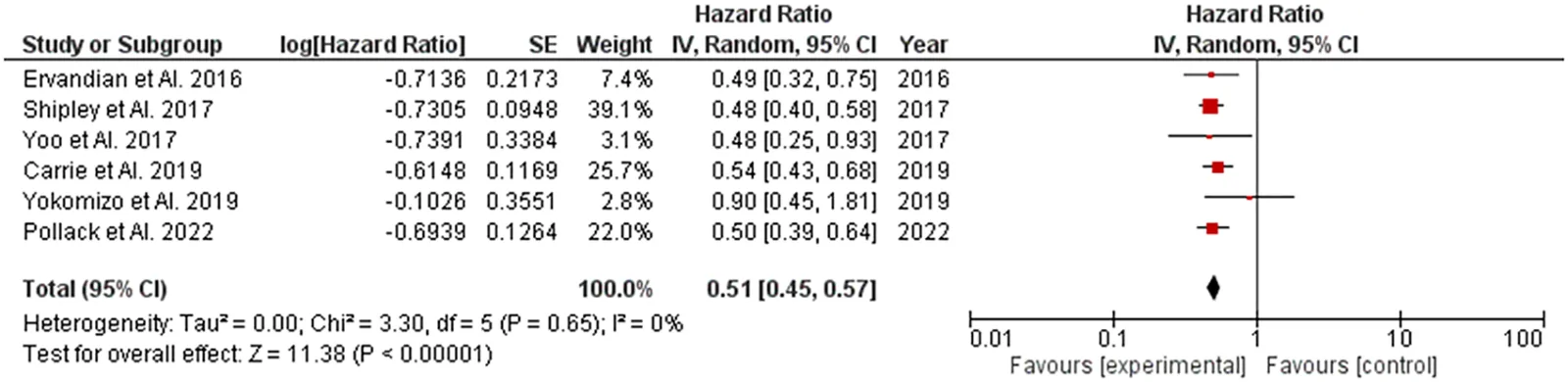
Meta-analysis of biochemical progression-free survival.

For biochemical progression-free survival (bPFS), Cortés-González et al. (2013) (29), Ervandian et al. (2016) (30), Yoo et al. (2017) (32) and Yokomizo et al. (2019) (23), reported that combined therapy (SRT plus ADT) or concomitant ADT was associated with improved bPFS (biochemical control rates of 60%–70% and HRs of 0.5–0.6 versus SRT alone or ADT alone), particularly among patients with high-risk features. Similarly, observational series (24, 25, 28) reported high bPFS rates at intermediate and long-term follow-up, whereas the Tendulkar et al. nomogram (2016) (31) confirms the key role of pre-SRT PSA, suggesting that the potential benefit of combined therapy is concentrated in patients with a higher residual disease burden. In the meta-analysis of six studies (**Figure 4**), the effect was more consistent and robust, showing a 49% relative reduction in the risk of biochemical recurrence (HR 0.51; 95% CI 0.45–0.57; p<0.00001) with no statistical heterogeneity (I²=0%), supporting a consistent beneficial effect on PSA control.

#### 3.2.3 Metastasis-free survival

Randomized trials suggest that combined therapy can improve MFS in selected settings. In Shipley et al. (2017) (21), the addition of 24 months of bicalutamide to SRT reduced the hazard of metastasis (HR 0.63; 95% CI 0.46–0.87; p=0.005), with 85.5% of patients remaining metastasis-free at 12 years. Similarly, Carrie et al. (2019) reported improved 10-year MFS with combined therapy compared with SRT alone (75% vs 69%; HR 0.73; 95% CI 0.54–0.98; p=0.034). In Parker et al. (2024) (26, 27) extending ADT duration from 6 to 24 months was associated with a lower risk of metastasis or death (HR 0.77; 95% CI 0.61–0.98; p=0.029) and higher 10-year MFS (78.1% vs 71.9%), whereas adding only 6 months of ADT to SRT did not yield a statistically significant benefit (HR 0.89; 95% CI 0.69–1.14; p=0.35). Yoo et al. (2017) (32) also reported higher 5-year MFS with combined therapy (100%) versus SRT alone (87.3%; p=0.021).

**Figure 5:**
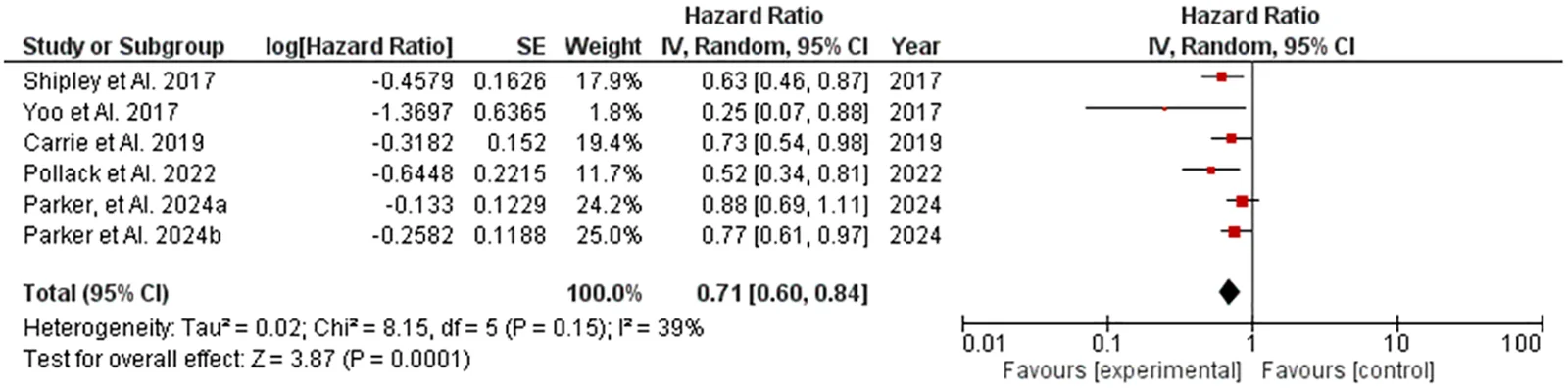
Meta-analysis of metastasis-free survival.

Observational studies by Elakshar et al. (2023) (25) and Cortés-González et al. (2013) (29), described prolonged metastasis-free survival and confirmed high-risk factors such as seminal vesicle invasion; however, their design limits causal attribution of the observed benefit to ADT. When these findings were incorporated into the meta-analysis of six trials (**Figure 5**), combined therapy was associated with a 29% relative reduction in the risk of developing metastases (HR 0.71; 95% CI 0.60–0.84; p=0.0001), with moderate heterogeneity (I²=39%) **Table 2**.

**Table 2:** Survival outcomes.

Author/Year	Type of survival	Follow-up (years)	Results
Ospino et Al. 2012	c-PFS	2,4	94%
Cortés-González et Al. 2013	OS / b-PFS	4	OS 95%. b-PFS 63%. SVI predictor of metastasis (p 0,007)
Ervandian et Al. 2016	b-PFS	3,1	Global: 57% 68% with ADT vs 47.8% without ADT (p<0.001).
Tendulkar et Al. 2016	b-PFS / MFS	5	b-PFS 56%, 71% (PSA ≤0,2) vs 37% (PSA >2) (p<0,001). MFS 90%
Shipley et Al. 2017	OS / b-PFS / MFS	12	OS: 76.3% vs 71.3% (HR 0.77; 95% CI 0.59–0.99; p=0.04). bPFS: SRT+ADT 44% vs SRT alone 32% (HR 0.48; 95% CI 0.40–0.58; p<0.001). MFS: 85.5% vs 77.0% (p=0.005).
Yoo et Al. 2017	OS / b-PFS / MFS	6	OS: RT alone 97.6% vs RT+ADT 98.5% (p=0.440). b-PFS: RT alone 62.9% vs SRT+ADT 68.3%. MFS: RT alone 87.3% vs SRT+ADT 100%.
Fossati et Al. 2019	c-PFS	9,4	c-PFS 92% (p 0,022)
Carrie et Al. 2019	OS / b-PFS / MFS	10	OS: SRT+ADT 86% vs SRT alone 85% (HR 0.93; 95% CI 0.63-1.39; p=0.73). bPFS: 5 years: SRT+ADT 80% vs SRT alone 62% (HR 0.56; 90% CI 0.40-0.77; p=0.001). bPFS: 10 years: SRT+ADT 64% vs SRT alone 49% (HR 0.54; 95% CI 0.43-0.68; p<0.0001). MFS: 75% (p=0.034).
Yokomizo et Al. 2019	OS / PFS	8,8	OS: 91.4% (p=0.94). PFS (5 years): 88.9% vs 93.8% (HR 0.90; 95% CI 0.45–1.81; p=0.77).
Pollack et Al. 2022	OS / PFS / MFS	8,2	OS: No difference (HR 0.98; p=0.917). PFS: 81% (RT+ADT) vs 87% (RT+N1+ADT). MFS: HR 0.52 (97.5% CI 0.34–0.81).
Elakshar et Al. 2023	OS / b-PFS / MFS	10	OS: 97.0%. bPFS: 68.3%. MFS: 72.9%
Parker et Al. 2024	OS / c-PFS / MFS	10	OS: 82.0% (short-term ADT) vs 84.6% (long-term ADT) (HR 0.88; 95% CI 0.663–1.169; p=0.38). c-PFS: 73.1% (long-term ADT) vs 66.5% (short-term ADT) (HR 0.73; 95% CI 0.59–0.90; p=0.0024). MFS: 71.9% (short-term ADT) vs 78.1% (long-term ADT).
Parker et Al. 2024	OS / c-PFS / MFS	10	OS: 85.3% (ADT 6 months) vs 85.6% (no ADT) (HR 0.88; 95% CI 0.65–1.19; p=0.42). c-PFS: 79.4% (HR 0.54; 95% CI 0.43–0.68; p<0.0001). MFS: SRT alone 79.2% vs ADT 6 months 80.4% (HR 0.886; 95% CI 0.688–1.140; p=0.35).

OS, overall survival; PFS, progression-free survival; c-PFS, clinical progression-free survival; b-PFS, biochemical progression-free survival; MFS, metastasis-free survival; PSA, prostate-specific antigen; SRT, salvage radiotherapy; ADT, androgen deprivation therapy; SVI, seminal vesicle invasion.

**Figure 6:**
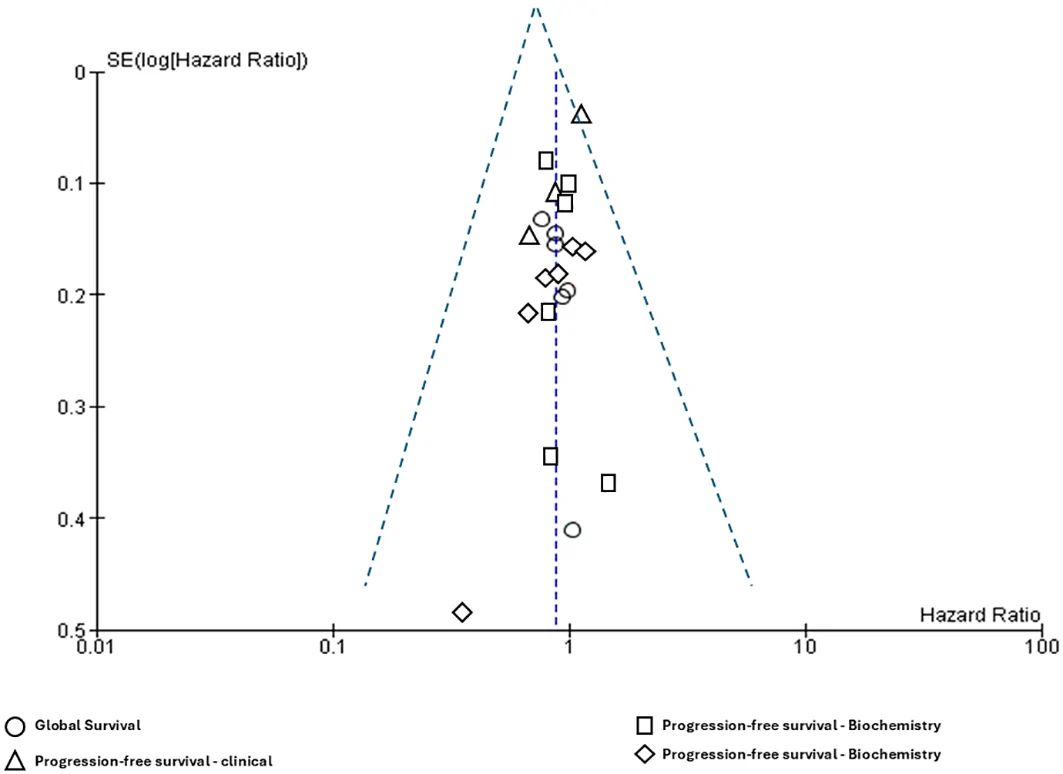
Funnel plot for survival outcomes.

The heterogeneity assessment is shown in **Figure 6**, which summarizes the main results of this meta-analysis (OS, b-PFS, c-PFS, and MFS). This funnel plot shows an overall symmetric distribution, not suggestive of publication bias.

### 3.3 Toxicity

In terms of safety, the combination of SRT and ADT showed an overall acceptable tolerability profile, with a predictable increase in endocrine-related adverse effects. Shipley et al. (2017) (21), reported a marked increase in gynecomastia with bicalutamide (70% vs 11%), without serious cardiovascular events.

Yokomizo et al. (2019) (23), observed that SRT was associated with gastrointestinal toxicity (proctitis 53%, rectal bleeding 34%), whereas hormone therapy accounted for most endocrine and sexual events (gynecomastia 91%; grade 3: 4%; erectile dysfunction 80%). Carrie et al. (2019) (22), documented late grade ≥3 genitourinary events in 8%, with a low frequency of severe events.

Across observational studies, toxicity was generally low, and quality of life was largely preserved, Cortés-González et al. (2013) (29), reported infrequent acute grade ≥3 events (genitourinary (GU) 3%) and moderate late toxicity at 5 years (GU 9%, gastrointestinal (GI) 5%), without clinically meaningful impairment in quality-of-life measures. In Elakshar et al. (2023) (25) Grade ≥ 2 ADT-related toxicity occurred in 10.8% of patients and decreased to 2.2% after treatment discontinuation. Ospino et al. (2012) (28)) reported toxicity in 35% of patients, with no relevant grade ≥ 3 events.

In randomized evidence, treatment intensification was associated with higher acute toxicity but limited severe events. Pollack et al. (2022) (24), found an increased rate of acute grade ≥ 2 adverse events (22.9%), whereas grade ≥ 3 events remained uncommon (1.2%); late toxicity was comparable between groups, except for a higher incidence of grade ≥ 2 hematologic events with combined therapy. In Parker et al. (2024) (26, 27) extending ADT duration to 24 months increased grade ≥ 3 toxicity (19% vs 14%; p=0.025), driven by urethral stricture and hematuria. By contrast, SRT alone versus SRT plus short-term ADT (6 months) yielded similar rates of grade ≥ 3 toxicity (17% vs 14%; p=0.15), and no treatment-related deaths were reported (**Table 3**).

**Table 3:** Toxicity outcomes.

Author/Year	Type of toxicity	Results
Ospino et Al. 2012	Acute GI and GU	GI: ≥G2: 7,5%; GU ≥G2: 5% ≥G3 0%
Cortés-González et Al. 2013	Acute/late	Acute toxicity ≥G2: 18% Late ≥G2: 10% ≥G3: 0%.
Ervandian et Al. 2016	GI and GU late	≥G2: GU 9%, GI 6%.
Shipley et Al. 2017	GU/GI	Endocrine (gynecomastia) 70%. GU/GI ≥G3 <5%.
Yoo et Al. 2017	GU/GI	≥G2: GU 8%, GI 6%.
Carrie et Al. 2019	GU/GI	≥G2: GU 15%, GI 11%.
Yokomizo et Al. 2019	GU/GI	≥G2: GU 12%, GI 8%. ≥G3 <3%.
Pollack et Al. 2022	GU/GI	Acute toxicity: grade ≥2, 22.9%; grade ≥3, 1.2%. Late toxicity: grade ≥2, 27.0%; grade ≥3, 2.3%.
Elakshar et Al. 2023	Acute/late GI and GU	Late toxicity (grade ≥2): GU 11%; GI 9%.
Parker et Al. 2024	GU/GI	≥G3 24m:19% vs 6m: 14%, (p 0,025).
Parker et Al. 2024	GU/GI	G3 ≥ toxicity: SRT alone 17% vs SRT+ADT (6 months) 14% (p=0.15).

GU, genitourinary; GI, gastrointestinal; ADT, androgen deprivation therapy; G3, grade 3.

### 3.4 Quality assessment of the included studies

**Figure 7:**
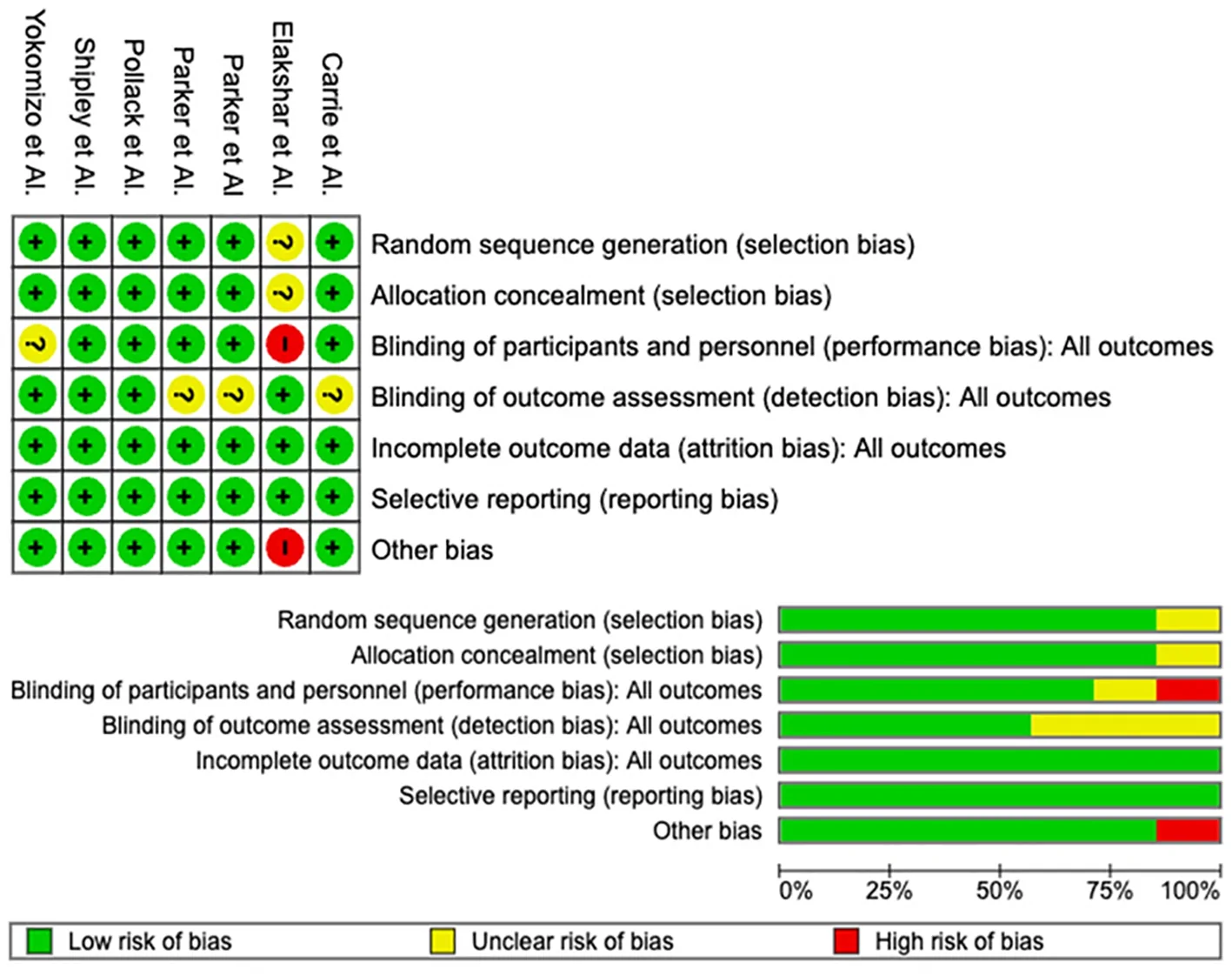
Risk of bias assessment of randomized trials using RoB 2.0.

The included randomized clinical trials Shipley et al. (2017) (21), Carrie et al. (2019) (22), Yokomizo et al. (2019) (23), Elakshar et al. (2023) (25), Pollack et al. (2022) (24) and Parker et al. (2024) (26, 27) were assessed using the RoB 2.0 tool (16) (**Figure 7**). The randomization process and allocation concealment were adequate, losses to follow-up were minimal and balanced between groups, outcomes were clearly and consistently defined, and there was no evidence of selective reporting. In contrast, Elakshar et al. (2023) (25) was a prospective, single-arm, phase II, non-randomized trial. Although operational blinding could not always be ensured, outcomes were objective, attrition was low, and no major sources of bias were identified in outcome measurement or reporting; however, the lack of random allocation entails inherent limitations.

**Figure 8:**
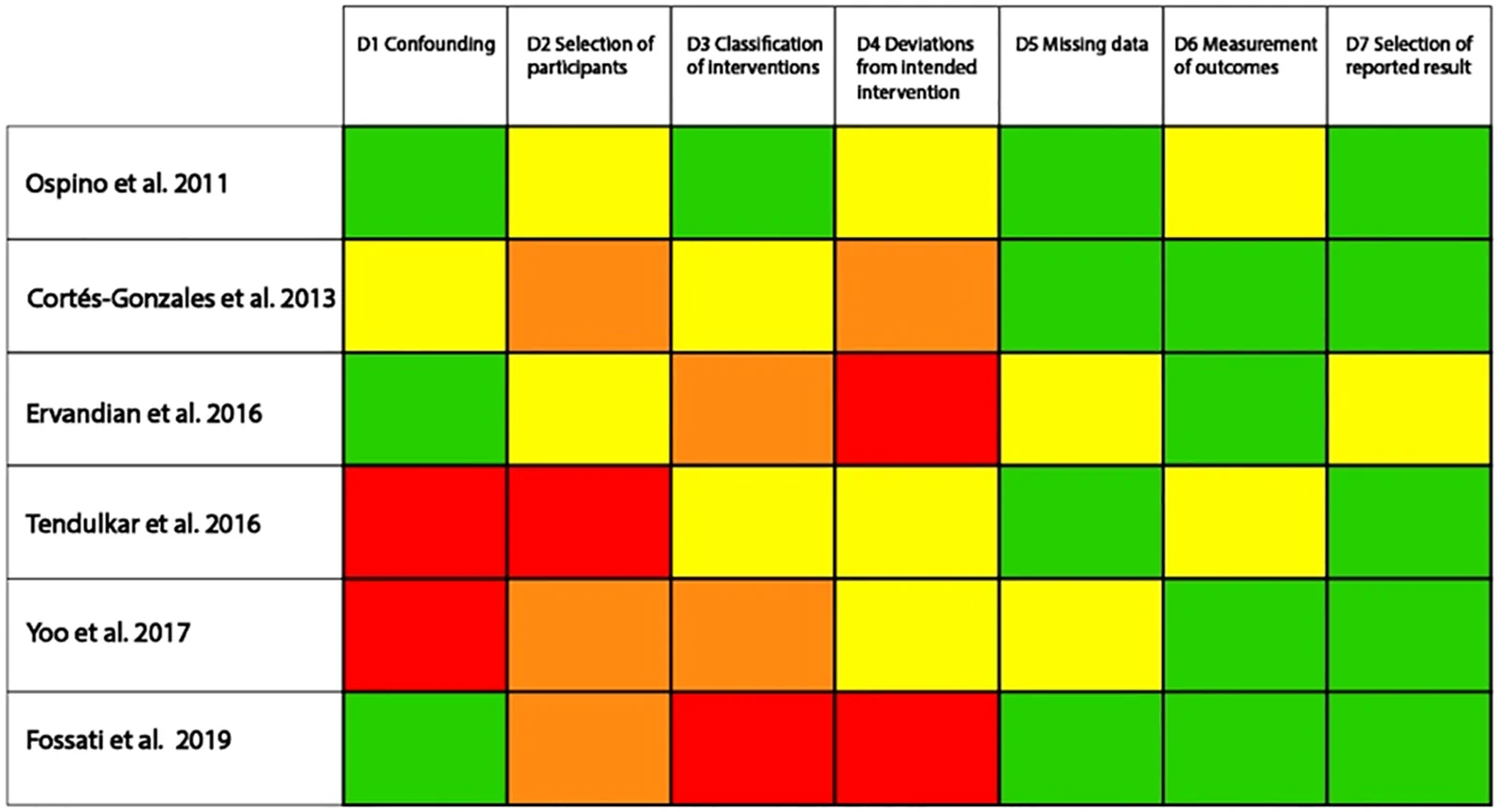
Traffic Light Plot. Risk of bias assessment of observational studies using ROBINS-I. Green: low risk. Yellow: moderate risk. Orange: severe risk. Red: critical risk.

For observational studies: Ospino et al. (28) (2012), Cortés-González et al. (2013) (29), Ervandian et al. (2016) (30), Tendulkar et al. (2016) (31) Yoo et al. (2017) (32) and Fossati et al. (2019) (33) risk of bias was assessed using the ROBINS-I tool (15) (**Figure 8**). Overall, the risk of bias was high, due to residual confounding and, to a lesser extent, variability in participant selection. Across the remaining domains, judgments were moderate to low, with no critical ratings. Taken together, the non-randomized studies pointed in the same direction of effect as the randomized trials.

## 4 Discussion

Salvage radiotherapy (SRT) emerged as a therapeutic strategy to control biochemical recurrence after radical prostatectomy in patients with localized prostate cancer (11). Clinical decision-making requires determining whether the therapeutic goal should be addressed with systemic therapy or a local approach. SRT directed to the prostate bed is a potentially curative option when the disease remains locally confined, particularly when initiated at low PSA levels. In contrast, androgen blockade provides systemic control and may enhance radiotherapy outcomes in selected subgroups, although it is not free of toxicity (35).

Early on, the indication for SRT was based primarily on PSA elevation in the absence of imaging confirmation of metastatic disease (36). Outcomes were variable, due to heterogeneity in patient selection criteria (37). Over time, evidence has shown that earlier initiation of SRT at low PSA levels is associated with improved oncologic outcomes, including better biochemical control and a reduced risk of clinical progression and metastasis (38).

The timing of biochemical recurrence is not clearly established in the literature. In this context, Monti et al. (2006) (39) provide relevant information by describing the temporal dynamics of recurrence after SRT: the mean time to recurrence was 15 months, with an overall recurrence rate of 39.3%. This finding highlights a critical window during the first years following SRT, when the highest risk of biochemical failure appears to concentrate. Clinically, however, some patients did not achieve a sustained oncologic control threshold, underscoring the potential importance of treatment intensification with ADT in patients with two or more high-risk factors (11).

Clinical trials and retrospective studies show a consistent pattern in which early SRT provides better oncologic control than ADT alone. SRT as monotherapy is a potentially curative strategy when delivered early in low- to intermediate-risk profiles, or in patients without adverse features such as PSA ≤0.5 ng/mL, pT2-pT3a stage, PSA doubling time (PSA-DT) >6 months, and Gleason score ≤7 (11, 40). Under these conditions, SRT alone achieves high 5-year biochemical control rates while avoiding the systemic toxicity associated with ADT (24). Across studies, outcomes were characterized by improved biochemical control and higher 10-year metastasis-free survival. However, freedom from failure decreased stepwise as pre-SRT PSA increased: 71% for PSA 0.01–0.20 ng/mL, 63% for 0.21–0.50 ng/mL, and 37% when PSA was >2 ng/mL.

These findings were supported by Song et al. (2010) (41), who added a key practical insight by showing that patients at low and intermediate risk may benefit from SRT alone. In their cohort, the most favorable outcomes were observed among patients with pre-SRT PSA ≤ 0.5 ng/mL, pathologic stage pT2, and Gleason score ≤ 7 (41). Overall, these characteristics are consistent with a localized pattern of recurrence and a favorable risk profile, reinforcing the concept that SRT alone may be curative even without ADT in low- and intermediate-risk patients, thereby avoiding the toxicity associated with hormone therapy (41).

Currently, SRT is recognized as a potentially curative intervention after radical prostatectomy, and available evidence supports greater efficacy when it is combined with androgen deprivation therapy (ADT) (37, 38). Contemporary practice has increasingly emphasized risk stratification, such that combined therapy for biochemical recurrence is reserved for high-risk scenarios, including pre-SRT PSA >0.5 ng/mL, PSA-DT <6 months, ISUP grade group 4-5, pathologic stage ≥pT3b, positive surgical margins, or persistent PSA after surgery (38, 42).

In our meta-analysis, adding ADT to SRT was associated with a 29% relative reduction in the risk of developing metastases (HR 0.71; 95% CI 0.60–0.84; p=0.0001; I²=39%) and an even more pronounced 49% relative reduction in the risk of biochemical recurrence (HR 0.51; 95% CI 0.45–0.57; p<0.00001; I²=0%). Consistently, when pooling outcomes of clinical progression or death, combined therapy reduced the relative risk by 28% (HR 0.72; 95% CI 0.53–0.98; p=0.03), although heterogeneity was high (I²=91%), likely driven by differences in baseline risk profiles, PSA thresholds at treatment initiation, and ADT duration across studies.

Intensification trials help refine *when* and *in whom* ADT should be added, rather than supporting indiscriminate use. Shipley et al. (21), showed that 24 months of ADT reduced the relative risk of death by 23%, with a 12-year OS of 76%; however, the benefit was concentrated among patients with pre-SRT PSA 0.7–1.5 ng/mL. By contrast, in those with PSA <0.7 ng/mL, no OS advantage was observed and non–cancer-related mortality increased, suggesting that intensification in low-risk settings may be unnecessary or potentially harmful.

Consistently, most randomized trials and observational cohorts showed approximately 50% reductions in progression risk among high-risk patients (22, 24, 30) with significant improvements in PFS and MFS when short- or long-term ADT was added. Fossati et al. (2019) (33) further demonstrated that the magnitude of benefit depends on the number of adverse factors, with the greatest benefit observed in patients with two or more high-risk features (Gleason score ≥ 8, pT ≥ 3b, and pre-SRT PSA >0.5 ng/mL). Therefore, risk stratification should guide the indication of ADT in combination with SRT, reserving its use for high-risk settings (33).

In contrast, the effect on overall survival did not reach robust statistical significance. Across studies, a relative reduction in the risk of death favored combined therapy; however, confidence intervals were wide (95% CI 0.76–1.00), reflecting limited statistical power due to a low number of events and clinical heterogeneity across studies. When the analysis was restricted to higher-risk subgroups (elevated pre-SRT PSA, Gleason score ≥8, pathologic stage ≥pT3b, positive surgical margins, or short PSA-DT) (10, 40), the effect became statistically significant, showing a clinically meaningful reduction in mortality risk that is consistent with individual trial findings. These results suggest that the contribution of ADT to overall survival is risk-dependent, supporting a selective approach in patients with biochemical recurrence and high-risk features rather than routine use across all SRT settings.

Taken together, these findings combined therapy as a treatment-intensification strategy with clinically meaningful benefit in the control of biochemical recurrence, and support its preferential use in patients with high-risk features, while reserving salvage radiotherapy (SRT) alone for low-risk profiles or for those in whom comorbidities limit exposure to androgen deprivation therapy (ADT). The available evidence supports improved biochemical an metastasis-related disease control with the addition of hormonal therapy to salvage radiotherapy, whereas the magnitude and consistency of the overall survival benefit remain uncertain and appear to vary according to baseline risk (40).

Funnel plot assessment did not reveal meaningful asymmetry, reducing the likelihood of substantial publication bias and strengthening the robustness of the findings. Collectively, these results support the preferential use of combined therapy in patients with biochemical recurrence and high-risk features following radical prostatectomy.

Importantly, the optimal duration of ADT remains uncertain. The available evidence suggests a risk-adapted benefit: patients with two or more adverse histopathological risk factors derive greater benefit from prolonged ADT (up to 36 months), whereas in patients with a single risk factor, short-course ADT (<12 months) appears sufficient (21–24). These findings further support the clinical value of individualized risk stratification when planning combined-modality treatment.

To inform decision-making in our local context, we considered the cohort reported by Ospino et al. (2012) (28). Although the study was not primarily designed to evaluate the effect of combined therapy, it provides a meaningful contribution by corroborating the clinical rationale for treatment intensification in biochemical recurrence and generating evidence from a Latin American setting. In this cohort of Colombian patients treated with salvage radiotherapy, the addition of ADT was associated with a significant reduction in the risk of biochemical progression, in keeping with findings from randomized clinical trials (26–28). Importantly, these data suggest that the benefits of combined therapy are reproducible across healthcare systems and populations beyond those represented in the original studies, thereby strengthening their applicability within our clinical setting.

In clinical practice, pre-SRT PSA should be interpreted in conjunction with Gleason score (≥ 8), pathological T stage (≥pT3b), surgical margin status, PSA doubling time (PSA-DT; <6–12 months), and time to recurrence. In low- to intermediate-risk patients with PSA ≤0.5 ng/mL, SRT alone remains the initial standard; however, when ≥ 2 adverse risk factors are present, the addition of ADT should be considered, with treatment duration tailored to the individual risk profile (33).

Regarding safety, most studies consistently indicate that adding ADT is not associated with severe toxicity when administered for predefined, time-limited periods (26). Nevertheless, an increased incidence of adverse effects (such as gynecomastia and erectile dysfunction) was observed, particularly with bicalutamide (21), although these events did not lead to treatment discontinuation. Cortés-González et al. (29), provided the most substantive contribution to the quality-of-life assessment, reporting that quality of life may remain stable with combined therapy, thereby supporting its clinical feasibility. However, the limited availability and heterogeneous reporting of adverse-event data across several included studies represent an important methodological limitation.

The relevance of this review lies in its comprehensive synthesis and critical appraisal of the most robust evidence currently available, thereby clarifying how SRT supports a clinical decision-making framework that increasingly prioritizes precision medicine. The analysis of clinical studies further provides evidence of applicability, showing that the benefits of therapeutic intensification are reproducible across diverse healthcare settings. Overall, this critical assessment informs the refinement of clinical recommendations and the optimization of management strategies for biochemical recurrence of prostate cancer, bridging global evidence with regional clinical practice.

Nevertheless, certain limitations must be considered in context. This study is limited by the inherent heterogeneity in the pelvic radiotherapy fields utilized across the included trials. Specifically, the pooling of prostate bed-only radiotherapy with whole pelvic radiotherapy—the latter typically reserved for higher-risk patients—may influence estimates of both toxicity and ADT efficacy. Due to the lack of granular patient-level data, a formal subgroup analysis was not feasible. Consequently, these findings should be interpreted with caution when applying them to distinct risk-stratified clinical cohorts.

Furthermore, the clinical trials included relied on conventional imaging modalities for baseline staging. Conventional imaging is well-known to underestimate microscopic disease burden, potentially leading to stage migration. Consequently, the apparent survival and recurrence benefits of whole-pelvic radiotherapy or extended ADT might be partially driven by the unintended treatment of occult metastatic disease. Given that modern clinical practice increasingly utilizes PSMA PET/CT for staging, which offers significantly higher sensitivity and specificity, the absence of PSMA-based staging in the primary literature limits the direct generalizability of our pooled estimates to contemporary patient cohorts.

Finally, a key limitation of our analysis is the simultaneous pooling of randomized trials and observational designs. Although the subgroup analyses mitigate this by isolating study types, the main combined results must be interpreted with caution. The observational data included herein should be interpreted as supportive and hypothesis-generating rather than confirmatory.

These considerations underscore the need for additional prospective studies to better define patient selection criteria, optimize oncologic outcomes, and more precisely determine the most appropriate combination and duration of therapies in recurrence of prostate cancer.

## 5 Conclusions

The combination of salvage radiotherapy (SRT) and androgen deprivation therapy (ADT) confers clinically meaningful benefit in patients with localized prostate cancer who develop biochemical recurrence after radical prostatectomy and are at high risk of progression, as defined by adverse features such as pre-SRT PSA >0.5 ng/mL, PSA doubling time (PSA-DT) < 6 months, ISUP grade group 4–5, pathological stage ≥pT3b, positive surgical margins, or persistent PSA following surgery. Across the included studies, combined therapy was consistently associated with improvements in biochemical progression-free survival (bPFS) and metastasis-free survival (MFS) compared with SRT alone; however, uncertainty remains regarding its impact on clinical progression-free survival (cPFS) and overall survival (OS). These findings underscore the importance of appropriate risk stratification when determining both the indication for ADT and its duration, prioritizing treatment intensification for patients with high-risk prognostic factors. Further studies with more homogeneous selection criteria, standardized outcome definitions, and longer follow-up are warranted to more precisely delineate the patient subgroups that derive the greatest benefit from combined-modality treatment.

## Data Availability

The raw data supporting the conclusions of this article will be made available by the authors, without undue reservation.
