# Supplementary material for: Use of androgen suppression therapy at salvage radiotherapy in recurrent prostate cancer: a systematic review with meta-analysis
Source: Front Urol. 2026 Aug 17;6:1836469. doi: 10.3389/fruro.2026.1836469 (PMC13526558; doi:10.3389/fruro.2026.1836469)
Supplement: Supplementary file 1 [file Supplementaryfile1.docx]

Supplementary Material

# 1 Supplementary Appendix 1. Database search strategy

Medline PubMed

Prostatic Neoplasms[MeSH Terms] OR ((Prostate[Title/Abstract] OR prostatic[Title/Abstract]) AND (Cancer*[Title/Abstract] OR neoplasm*[Title/Abstract]))) AND (Recurrence[MeSH Terms] OR Neoplasm Recurrence, Local[MeSH Terms] OR Recurrence*[Title/Abstract] OR Relapse*[Title/Abstract]) (Radiotherapy[MeSH Terms] OR radiotherapy[Title/Abstract] OR Brachytherapy[MeSH Terms]) AND (Salvage[Title/Abstract] OR rescue[Title/Abstract]).

EMBASE

('prostate cancer'/exp OR 'prostatic neoplasm':ti,ab) AND ('cancer recurrence'/exp OR 'biochemical recurrence':ti,ab OR 'PSA relapse':ti,ab) AND ('salvage therapy'/exp AND 'radiotherapy'/exp OR 'salvage radiotherapy':ti,ab) AND ('androgen deprivation therapy'/exp OR 'hormone therapy'/exp OR 'androgen suppression therapy':ti,ab) AND ('overall survival'/exp OR 'progression free survival'/exp OR 'treatment outcome'/exp OR 'disease free survival':ti,ab).

SCOPUS

(Prostatic Neoplasms[MeSH Terms] OR ((Prostate[Title/Abstract] OR prostatic[Title/Abstract]) AND (Cancer*[Title/Abstract] OR neoplasm*[Title/Abstract]))) AND (Recurrence[MeSH Terms] OR Neoplasm Recurrence, Local[MeSH Terms] OR Recurrence*[Title/Abstract] OR Relapse*[Title/Abstract]) (Radiotherapy[MeSH Terms] OR radiotherapy[Title/Abstract] OR Brachytherapy[MeSH Terms]) AND (Salvage[Title/Abstract] OR rescue[Title/Abstract]).

LILACS

(mh:(Neoplasias de la Próstata) OR próstata OR prostático OR cáncer* OR neoplasia*) AND (mh:(Recurrencia Local de Neoplasia) OR recurrencia* OR recaída*) AND (mh:(Radioterapia) OR radioterapia OR mh:(Braquiterapia)) AND (salvamento OR rescate).

CENTRAL

("prostate cancer" OR "prostatic neoplasm") AND ("biochemical recurrence" OR "PSA relapse") AND ("salvage radiotherapy") AND ("androgen deprivation therapy" OR "hormonal therapy") AND ("overall survival" OR "progression-free survival").
